# Recent Advances in MXene Nanocomposite-Based Biosensors

**DOI:** 10.3390/bios10110185

**Published:** 2020-11-20

**Authors:** Jinho Yoon, Minkyu Shin, Joungpyo Lim, Ji-Young Lee, Jeong-Woo Choi

**Affiliations:** 1 Department of Chemical & Biomolecular Engineering, Sogang University, 35 Baekbeom-Ro, Mapo-Gu, Seoul 04107, Korea; moonchild@sogang.ac.kr (J.Y.); mkshin91@sogang.ac.kr (M.S.); jpim92@sogang.ac.kr (J.L.); 2 Department of Chemistry and Chemical Biology, Rutgers, The State University of New Jersey, Piscataway, NJ 08854, USA

**Keywords:** biosensors, MXenes, nanomaterials, high sensitivity/selectivity, electrochemical biosensor, fluorescent/optical biosensor

## Abstract

The development of advanced biosensors with high sensitivity and selectivity is one of the most demanded concerns in the field of biosensors. To meet this requirement, up until now, numerous nanomaterials have been introduced to develop biosensors for achieving high sensitivity and selectivity. Among the latest nanomaterials attracting attention, MXene is one of the best materials for the development of biosensors because of its various superior properties. MXenes are two-dimensional inorganic compounds with few atomic layers that possess excellent characteristics including high conductivity and superior fluorescent, optical, and plasmonic properties. In this review, advanced biosensors developed on the basis of the MXene nanocomposite are discussed with the selective overview of recently reported studies. For this, introduction of the MXene including the definition, synthesis methods, and its properties are discussed. Next, MXene-based electrochemical biosensors and MXene-based fluorescent/optical biosensors are provided, which are developed on the basis of the exceptional properties of the MXene nanocomposite. This review will suggest the direction for use of the Mxene nanocomposite to develop advanced biosensors with high sensitivity and selectivity.

## 1. Introduction

The development of biosensors to detect biological and chemical molecules that affect disease or have harmful effects is a major field of study [1,2]. The most important consideration in biosensor development is the accurate and rapid detection of target molecules to prevent the occurrence of diseases and to facilitate early medical treatment and thereby enhance the curative efficiency [3]. For this purpose, high sensitivity and selectivity are the most important characteristics to be considered in the development of biosensors. With the recent COVID-19 pandemic, the importance of biosensors in reducing the spread of diseases and facilitating treatment has become especially pertinent [4,5].

To achieve this goal, numerous nanomaterials ranging from metal nanoparticles and graphene to, more recently, transition metal dichalcogenide (TMD) nanomaterials have been studied and applied for the development of biosensors [6,7]. Metal nanoparticles have been utilized mostly in the past [8,9]. However, with the discovery of carbon nanomaterials such as carbon nanotubes and graphene, they have shown better performance than conventional metal nanoparticles [10,11]. Moreover, the biocompatibility makes them suitable for cellular state monitoring [12].

Nevertheless, the constant demand for nanomaterials with better performance and exceptional properties has led to the development of novel nanomaterials applicable for biosensors. Two-dimensional (2D) nanomaterials have attracted much interest because of their unique chemical, physical, and electrical properties [13]. In this regard, TMD nanomaterials such as molybdenum disulfide (MoS_2_) and tungsten disulfide (WS_2_) have been extensively studied for utilization as biosensors, and are considered to be the “beyond graphene” nanomaterials [14,15,16,17].

As such, various nanomaterials have been reported and utilized for the development of incrementally efficient biosensors. Among the most recently reported nanomaterials available for biosensors, MXenes have attracted much attention for their huge potential in biosensor development because of their characteristics [18,19]. MXenes are two-dimensional inorganic compounds with a thickness of a few atomic layers and are composed of transition metal carbides, nitrides, or carbonitrides such as titanium carbide (Ti_3_C_2_) and titanium carbonitride (Ti_2_CN), which confers them with exceptional characteristics, including high conductivity and superior fluorescent, optical, and plasmonic properties. [20,21,22]. Just as graphene and carbon nanotubes (CNTs) received great attention as new nanomaterials in the past, MXenes have been receiving much attention recently. MXenes represent a new novel material that has been recently used in various fields, such as catalyst generation, energy storage, and biosensors, due to their exceptional properties, including their excellent electrical conductivity, and semiconductor properties [23,24]. Moreover, the biocompatible property of MXenes enables their biomedical application [25,26,27]. Since they were first reported in 2011, MXenes have been used to develop various types of advanced biosensors, including electrochemical, fluorescent/optical, and surface-enhanced Raman spectroscopy (SERS) biosensors, by augmenting MXene characteristics to make them suitable for specific types of biosensors or by combining them with other nanomaterials [28,29,30]. Recent studies on the development of highly effective MXene biosensors show that this novel nanomaterial is the most ideal candidate for biosensor development at present.

**Figure 1:**
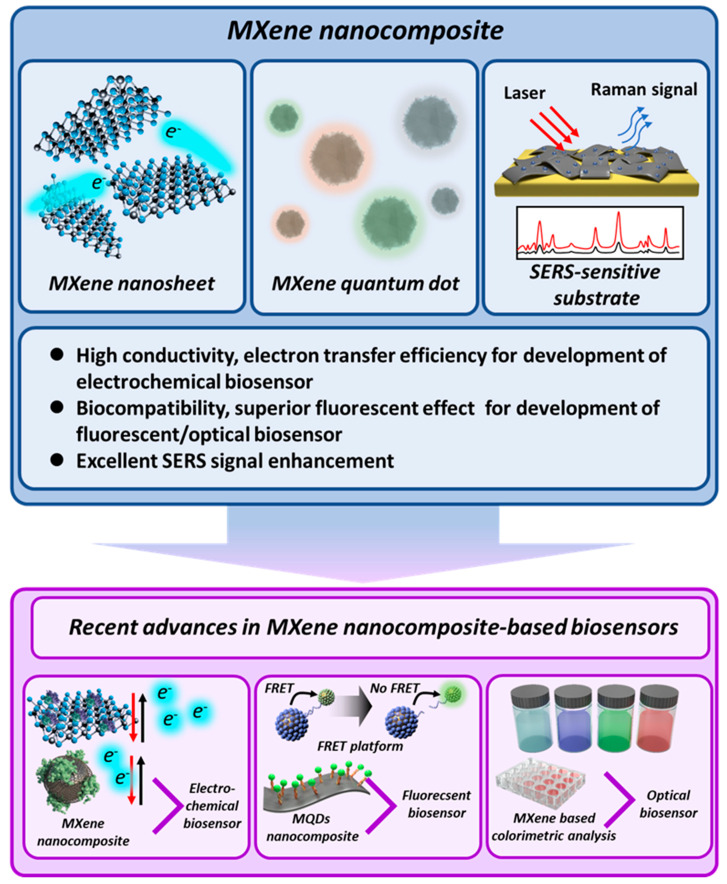
Recent advances in the development of advanced biosensors based on MXene nanocomposites.

In this review, we discuss the advanced biosensors developed on the basis of the exceptional properties of MXene nanocomposites and provide a selective overview of recently reported studies. More specifically, this review is divided into three topics: definition and characteristics of MXene, MXene-based electrochemical biosensors, and MXene-based fluorescent/optical biosensors (Figure 1). Accordingly, this review provides the synthesis methods and characteristics of various types of MXene and their unique advantages suitable for developing specific types of biosensors clearly. In conclusion, we strongly believe that this review can clarify the current research direction and methods for utilization of MXene nanocomposites to efficiently develop efficient biosensors with high sensitivity and selectivity.

## 2. Definition and Characteristics of MXenes

In this section, we define MXenes, describe their unique characteristics in the context of biosensor development, and present a widely used synthesis method.

### 2.1. Definition of MXenes

An MXene is essentially a 2D material consisting of transition metal carbides, carbonitrides, and nitrides, with a general formula of M_n+1_X_n_. Here, the M represents a transition metal such as titanium, zirconium, vanadium, tantalum, chromium, or molybdenum, while X represents carbon or nitrogen. Briefly, to synthesize MXenes, the MAX phase is used as the template material. The MAX phase, which has a general formula of M_n+1_AX_n_, has a sandwich structure with octahedral M_n+1_X_n_, and this structure shows strong M–X bonds and relatively weak M–A bonds. As precursors of MXenes, more than 70 different MAX phases have been identified, through which more than 20 MXenes can be synthesized [31,32]. The MXene is synthesized by removal of the A element from the MAX phase (M_n+1_AX_n_), which is usually Al in the MAX phase, using hydrofluoric acid (HF), hydrochloric acid–lithium fluoride salts (HCl-LiF), or hydrochloric acid–sodium fluoride (HCl-NaF) [33,34]. Accordingly, the MAX phase shows a strong interaction in comparison with other 2D materials such as graphene and TMD materials, including MoS_2_ and WS_2_. Moreover, unlike M–X bonds, which contain covalent, metallic, and ionic bonds, the M–A bonds contain only metallic bonds and can be selectively removed due to differences in their bonding strengths [35,36]. For this, the MAX phase was treated with etching reagents for the selective etching of the A element. As summarized in Table 1, MXenes can be synthesized with this etching treatment. In later sub-sections, the synthesis methods and the characteristics of MXenes are discussed in detail.

### 2.2. Synthesis Method and Characteristics

#### 2.2.1. HF Etching Method-Based MXenes and Characteristics

**Figure 2:**
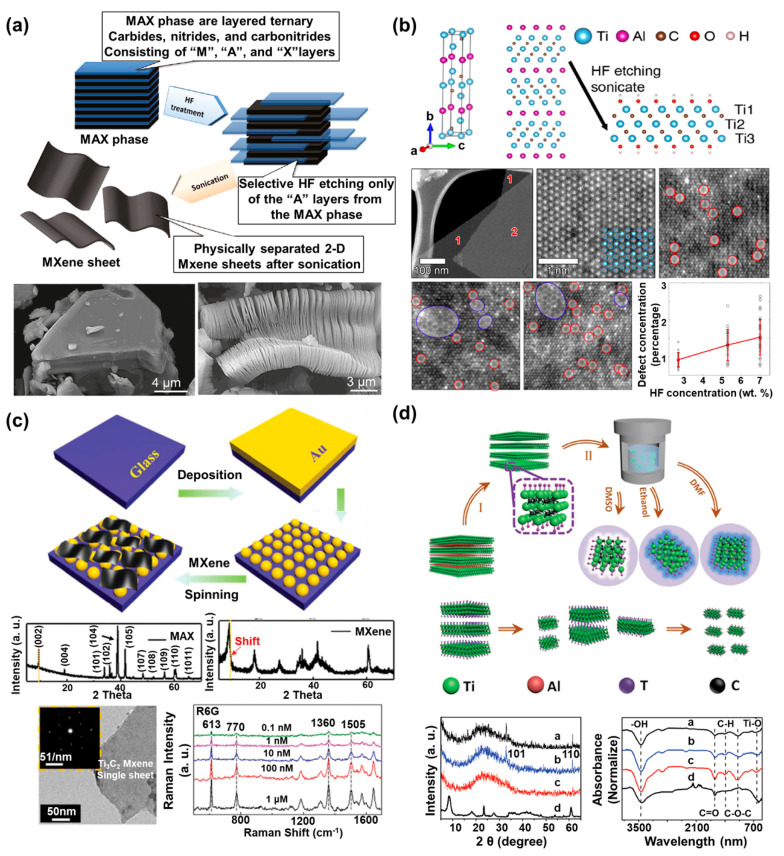
Definition and characteristics of MXenes. (**a**) Schematic diagram of the synthesis process from the MAX phase to the MXene nanosheets, and SEM images of the MAX phase (Ti_3_AlC_2_) before and after hydrofluoric acid (HF) treatment (reprinted with permission from [37]; copyright (2012) American Chemical Society). (**b**) Crystal structure images of the MAX phase of Ti_3_AlC_2_, and the low-magnification and atomic resolution high-angle annular dark field (HAADF)-scanning transmission electron microscopy (STEM) images of Ti_3_C_2_T_x_ (reprinted with permission from [39]; copyright (2016) American Chemical Society). (**c**) Schematic image of the MXene/Au nanostructure (NS) SERS substrate fabrication process, XRD pattern, and TEM image of synthesized MXene (Ti_3_C_2_T_x_), and the Raman spectra of R6G on the MXene/Au NSs SERS substrate with different concentration ranges of R6G (reprinted with permission from [45]; copyright (2019) The Royal Society of Chemistry). (**d**) Schematic image of the MXene-derived quantum dot (MQD) synthesis process using different solvents such as the dimethylformamide (DMF), DMSO, and ethanol (f-MQDs, s-MQDs, and e-MQDs, respectively), and the XRD pattern and FTIR spectra of the e-MQDs (black), f-MQDs (blue), and s-MQDs (red) (reprinted with permission from [41]; copyright (2018) John Wiley and Sons, Inc.).

With the increasing attention on the potential applicability of MXenes, researchers have studied various synthesis methods in order to develop MXenes more efficiently and to generate MXenes with superior properties. The first synthesis method used HF as the etching reagent to develop the MXene [37]. To synthesize MXenes by using HF, researchers immersed Ti_3_AlC_2_ powder, the MAX phase precursor, into 50% HF at room temperature for 2 h. The aluminum (Al) of the Ti_3_AlC_2_ powder was selectively etched with HF treatment, after which the functional groups, including oxygen (-O), hydroxyl (-OH), or fluorine (-F), were terminated on the surface of the MXene while the Al was removed to achieve thermodynamic stability. The suspension of the reacted solution was centrifuged several times with deionized (DI) water to precipitate the powder. As shown in Figure 2a, in comparison with the structure of the Ti_3_AlC_2_ powder before the HF treatment, the MXene synthesized by the HF treatment had a multilayer structure. In addition to this study, Nb_2_CT_x_ MXene nanosheets with a thickness of a few layers have been developed through control of the etching time by HF [38]. For this, the Nb_2_AlC powder was selected as a MAX phase precursor, and the Nb_2_AlC powder was immersed into 40% HF acid aqueous solution. After HF treatment, few-layer Nb_2_CT_x_ (f-Nb_2_CT_x_) and accordion-type multilayer Nb_2_CT_x_ (m-Nb_2_CT_x_) were fabricated by controlling the etching time. For f-Nb_2_CT_x_, the etching reaction was performed under 60 °C for 90 h, and the reacted solution was washed by centrifugation at 9000 rpm until the pH value of the supernatant became about pH 5 by changing of the solution with DI water. In addition, the supernatant was centrifuged at 5000 rpm for 5 min to obtain a pure precipitate. On the other hand, for m-Nb_2_CT_x_, all processes were performed in the same manner as for f-Nb_2_CT_x_, except that the etching time was reduced to 70 h. To confirm the electrochemical properties of the synthesized f-Nb_2_CT_x_ and m-Nb_2_CT_x_, the researchers used 1 M LiPF_3_ mixed with ethylene carbonate/dimethyl carbonate/ethylmethyl carbonate (EC/DEMC/EMC) as an electrolyte. The f-Nb_2_CT_x_ showed a high specific capacity of 354 mAh/g at 0.05 A/g after 110 cycles, in comparison with the m-Nb_2_CT_x_. This study verified that the structure and electrochemical properties of the synthesized MXene could be controlled well by decreasing the etching time.

MXenes synthesized using HF as the etching solution show excellent electrical, mechanical, and optical properties beyond those of the other 2D nanomaterials, including TMD and graphene. In particular, the electrical properties of MXenes are affected by functionalization of the surface during the etching process of MXene synthesis, and these surface properties confer high ion transport efficiency and excellent conductivity to the synthesized MXene [42,43]. In addition, the pristine MXene (M_n+1_X_n_) is metallic in nature due to the transition metal M, but as the surface becomes functional, it shows semiconducting properties. Moreover, MXenes have excellent biocompatibility and toxicity that may exist can be prevented because of its easy surface functionalization [25,26,27]. These unique electrical properties and biocompatibility of MXenes indicate their huge potential for use in the development of advanced electrochemical biosensors and biomedical application [44].

#### 2.2.2. In situ HF Etching Method-Based MXenes and Characteristics

Although the HF etching method is widely used for MXene synthesis, this method has some disadvantages such as the hazardous chemistry, requiring further treatment for monolayer morphology. Accordingly, other studies have suggested various approaches to compensate for the disadvantages of the HF etchant and presented synthesis methods using the HF etchants introduced thus far [18,46]. For example, one study described the development of single-layer Ti_3_C_2_T_x_ MXene with an atomic-scale defect structure [39]. In that study, single-layer Ti_3_C_2_T_x_ MXene was synthesized using the minimally intensive layer delamination (MILD) method, which eliminated the need for processes such as sonication, and large-size Ti_3_C_2_T_x_ MXene was synthesized using LiF in 6 M HCl. The different point defects of the synthesized Ti_3_C_2_T_x_ MXenes were confirmed by aberration-corrected atomic-resolution scanning transmission electron microscopy (STEM). Moreover, high-angle annular dark field (HAADF)-STEM imaging showed that the Ti vacancies were formed on the two-surface Ti sublayer, and that these defects were controlled by the concentration of HF used for the etching process (Figure 2b). Moreover, the conductivity of the synthesized single-layered Ti_3_C_2_T_x_ MXene was approximately 6.76 × 10^5^ S/m, which was comparable to that of metallic 1T-phase MoS_2_ (8 × 10^3^ S/m) or graphene (6 × 10^6^ S/m), which have been recognized as extremely conductive nanomaterials. In another study, 2D MXene Ti_3_C_2_ was developed by using bifluoride (NaHF_2_, KHF_2_, NH_4_HF_2_) [40]. To synthesize the Ti_3_C_2_ MXene, the researchers used TiH_2_, Al, and TiC (ratio of 1:1.1:2) as the initial powders. Next, the mixed powders were ball-milled for 12 h and reacted with an argon atmosphere at 1400 °C for 2 h to obtain a high-purity Ti_3_AlC_2_ block. Subsequently, 1 g of the synthesized Ti_3_AlC_2_ block was immersed into 1M bifluoride solution, which was the etching solution, at 60 °C for at least 8 h. In comparison with other synthesis methods that used the complex multi-step processes, this one-step process for synthesis of Ti_3_C_2_ by using bifluoride showed large interplanar spacing.

Although the properties of MXene prepared by in situ HF etching are similar to the method synthesized using HF, this MXene has advantages compared to the MXenes prepared by HF etching method such as the prevention of toxicity in the synthesis process and the structure of synthesized MXene being a monolayer. In addition, a method of expanding the SERS characteristics of MXene synthesized using in situ HF etching has been recently studied for the development of the SERS-sensitive electrodes. For example, a novel MXene nanosheet/Au nanostructure architecture (MXene/Au NSs) with high sensitivity and stability was developed for SERS-sensitive substrate fabrication [45]. In that study, to fabricate the SERS-sensitive substrate, the researchers deposited Au nanostructures (Au NSs) by thermal evaporation on the ultrasonically cleaned glass substrate at a rate of 0.1 nm/s under 7 × 10^−4^ Pa, and the deposited substrate was annealed at 600 °C for 900 s. Then, the MXene nanosheet (Ti_3_C_2_T_x_) was synthesized by the MILD method. The MXene/Au NS SERS substrate was fabricated by using layer-by-layer spin-coating of Ti_3_C_2_T_x_ at 800 rpm for 50 s on an Au NS substrate (Figure 2c). Next, Rhodamine 6G, a Raman-active probe, was employed on the MXene/Au NSs to verify the SERS-enhancing effect of the prepared substrate. The fabricated SERS-sensitive substrate showed 3.5-fold greater light average absorption, and the Raman intensity at 532-nm laser excitation showed a higher Raman signal peak in the presence of the MXene. On the basis of these characteristics, this SERS-sensitive substrate yielded a substantial enhancement factor of 2.9 × 10^7^ at an ultra-low concentration of 10^−10^ M. In addition, the fabricated SERS-sensitive substrate showed the stability for Raman signal detection over 20 days. Other studies have also attempted to develop various SERS-sensitive substrates by using MXenes modified with other materials such as the Au nanorod and Au–Ag nanoshuttles for signal enhancement [30,47].

#### 2.2.3. Synthesis of MQDs and Characteristics

In general, MXene has been synthesized as a multilayer or monolayer in the form of nanosheets, but recently, studies are being conducted to grant the superior optical property by synthesis of MXene in the form of quantum dots (QD). Moreover, similar to the other QD materials (TMD-based QDs, carbon-based QDs, and metal-based QDs), MXene-derived QDs (MQDs) exhibit superior optical properties such as strong fluorescence, high dispersibility, and biocompatibility [48]. In addition, recently, synthesis of MQDs as well as 2D nanosheet type MXene was reported by using the Ti_3_C_2_T_x_ MXene nanosheet powder [41]. The Ti_3_C_2_T_x_ MXene nanosheet powder was mixed with different solvents, including dimethylformamide (DMF), ethanol, or dimethyl sulfoxide (DMSO), under flowing N_2_ gas for 30 min to eliminate the oxygen. In addition, the mixture was hydrothermally reacted at 120 °C for 6 h, and, subsequently, the synthesized MQDs were collected by using centrifugation at 12000 rpm for 30 min (Figure 2d). In comparison with general MXene nanosheets without fluorescence properties, the synthesized MQDs showed different photoluminescence (PL) properties at 365 nm UV irradiation depending on the used solvents. The MQDs dissolved in DMSO showed white PL emission, and the MQDs in ethanol and DMF showed blue PL emission. Using the various MXene synthesis methods described here and in further studies, we expect that MXenes with various forms and properties will be synthesized using increasingly efficient and simple processes, and that these MXenes will find use in various scientific fields, including biosensors.

As discussed in this section, MXenes can be synthesized in various types and structures by different synthetic methods, and the synthesized MXenes show superior electrochemical, fluorescent/optical, and SERS signal-enhancing properties. Thus, MXenes show a huge potential for development of highly sensitive biosensors. Accordingly, we discuss the MXene-based electrochemical and fluorescent/optical biosensors in later sections.

## 3. MXene-Based Electrochemical Biosensors

Among the various types of electrochemical biosensors currently in use, enzymatic electrochemical biosensors have been studied extensively because the metalloenzymes widely used as sensing probes have redox properties that are suitable for electrochemical investigations [49,50]. However, the inherent characteristics of metalloenzymes, including their low electrochemical signal generation and instability, cause limitations in achieving high sensitivity and selectivity. To solve these problems, researchers have used MXenes to develop efficient enzymatic electrochemical biosensors, as summarized in Table 2. Since MXenes show exceptional characteristics, including high conductivity and electrochemical activity and a large surface area, they are being extensively studied for their application in the development of electrochemical biosensors. Thus, MXene-based electrochemical biosensors are reported more frequently than other types of MXene-based biosensors.

Recently, an electrochemical phenol biosensor based on MXene and tyrosinase has been reported and was used as the sensing probe for phenol detection [57]. Another study reported an enzymatic beta-hydroxybutyrate biosensor based on an enzyme-decorated MXene nanosheet [58]. In these biosensors, MXene nanosheets of the Ti_3_C_2_T_x_ type were synthesized and used as an effective immobilization matrix for beta-hydroxybutyrate dehydrogenase (beta-HBD), which was used as the sensing probe. To develop this biosensor, MXene was combined with beta-HBD by sonication for entrapping the beta-HBD. Meanwhile, bovine serum albumin (BSA) and glutaraldehyde (GA) were added to enhance beta-HBD stabilization through effective linkage. Next, a prepared composite composed of MXenes and beta-HBD was immobilized on the ruthenium hexamine-modified Au-printed circuit board electrode (AuPCB) by the drop-casting method. Ruthenium hexamine was introduced as the redox generator for demonstration of electrochemical detection of beta-hydroxybutyrate. The addition of beta-hydroxybutyrate to this biosensor allowed for controlling of the redox state of the ruthenium hexamine through the cascade reaction resulting from the interaction between beta-HBD and beta-hydroxybutyrate. The proposed biosensor detected beta-hydroxybutyrate with a high sensitivity (detection limit: 44.5 μM) and a wide linear response range (360 μM to 17.91 mM). Moreover, prepared biosensor retained its sensing property over 30 days (97.08% and 93.20% retention of its initial response after 7 and 30 days, respectively). This excellent long-term stability was derived from the layered structure of prepared MXene nanocomposite, which provided the more inner space between the BSA and the beta-HBD and provided the protective microenvironment. The results provided further evidence for the potential of MXene as an effective matrix for enzyme immobilization to construct the electrochemical biosensors.

**Figure 3:**
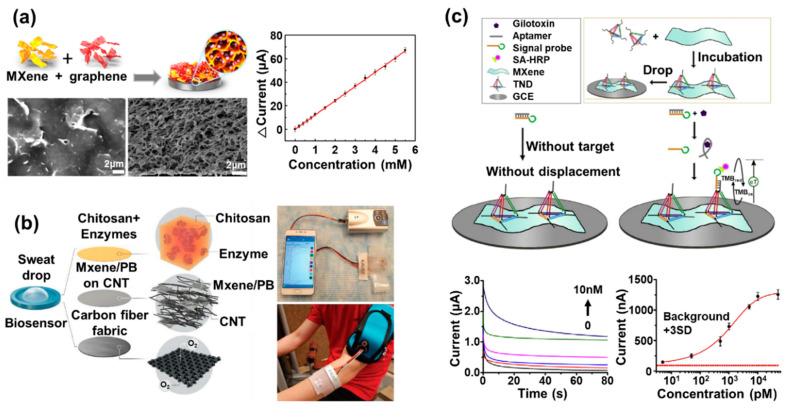
MXene-based electrochemical biosensors. (**a**) Construction of the MXene (Ti_3_C_2_T_x_) and graphene hybrid film (MG hybrid film): the SEM and cross-sectional images of the MG hybrid film, and the calibration plot of current versus different concentrations of the glucose obtained by using the MG hybrid film-based glucose biosensor (reprinted with permission from [51]; copyright (2019) American Chemical Society). (**b**) Schematic image of the sensing probe electrode in the wearable biosensing system based on the MXene/Prussian blue composite, and the optical image of the wearable electrochemical multifunctional biosensing patch system connected to the portable device and human body (reprinted with permission from [52]; copyright (2019) John Wiley and Sons, Inc.). (**c**) Schematic diagram of the label-free electrochemical gliotoxin biosensor based on the nanocomplexes composed of the tetrahedral DNA nanostructure (TDN), MXene nanosheet, and horseradish peroxidase (HRP); its sensing mechanism; and amperometric response curves, and the analyzed logarithmic plot for the detection of gliotoxin (reprinted with permission from [53]; copyright (2019) Elsevier publishing).

In other studies, the Ti_3_C_2_ MXene nanosheet was synthesized by the MILD method and its surface was biofunctionalized with aminosilane to provide covalent binding sites for immobilization of a sensing probe capable of detecting the cancer biomarker (carcinoembryonic antigen, CEA) [59]. The anti-CEA bioreceptor and hexaammineruthenium ([Ru(NH_3_)_6_]^3+^) were used as the sensing probe and the redox probe, respectively. Through the detection of CEA by the covalently bound sensing probe on the Ti_3_C_2_ MXene nanosheet, the redox signals from the ([Ru(NH_3_)_6_]^3+^) decreased due to the formation of the immune complex on the Ti_3_C_2_ MXene nanosheet, which was used to evaluate the detection of the CEA. Furthermore, covalent immobilization of the sensing probe induced uniform sensing probe layer formation through efficient surface modification of the Ti_3_C_2_ MXene nanosheet, and this system finally allowed for highly sensitive detection of the target biomarker (limit of detection: 1.0 nmol/L and linear response from 0.003 μM/L to 20.0 μM/L) and long-term stability of the proposed biosensor for 7 days. Moreover, in addition to the use of MXene alone for electrochemical biosensors, hybridization of MXenes with other suitable nanomaterials has been studied to develop novel electrodes [60,61]. The three-dimensional MXene (Ti_3_C_2_T_x_) and graphene hybrid film (MG hybrid film) was proposed to develop an electrochemical glucose biosensor [51]. To enhance the enzyme loading capacity and to avoid undesirable aggregation of the graphene, MXene and graphene were combined on the glassy carbon electrode to develop a three-dimensional porous film that provided effective support for enzyme immobilization (Figure 3a). By controlling the weight ratio of MXene and graphene, the researchers optimized the diameter of the pore in the MG hybrid film to facilitate enzyme loading inside the MG hybrid film. Glucose oxidase (GOx), the sensing probe for glucose, was introduced, which could react with glucose through a redox reaction. The prepared MG hybrid film showed excellent GOx-loading capacity because of its large activated surface area, abundant pores, and surface hydrophilicity. Moreover, it possessed outstanding electrical conductivity via exhibition of apparently enhanced redox peaks derived from the immobilized GOx on the MG hybrid film in comparison with other electrodes composed of only MXene or only graphene, which were used as controls. The prepared biosensor showed remarkable biosensing performance for detection of glucose diluted in real samples, with high sensitivity through the cyclic voltammetry investigation. This biosensor showed the 12.10 μA/mM sensitivity, which was more sensitive than the other types 3D porous material-based biosensors (e.g., 3D graphene film: 1.63 μA/mM), and it showed a good linear response range from 0.2 to 5.5 mM glucose. Moreover, the prepared biosensor showed the negligible decrease of the response current values over 300 scanning cycles, which was remarkably stable in comparison to the other control biosensors prepared with only MXene or only graphene. Moreover, numerous other major studies on MXene-based enzymatic electrochemical biosensors are currently underway [20].

Wearable biosensors have attracted great attention with the development of smart devices such as smart watches, and MXenes show potential for the development of wearable electrochemical biosensors [52]. Recently, a wearable electrochemical multifunctional biosensing patch system was reported on the basis of the MXene/CNT/Prussian blue composite on the flexible carbon fiber electrode to detect glucose, lactate, and hydrogen peroxide (H_2_O_2_) within sweat (Figure 3b). One of the most important achievements of this study was that detection of target molecules was conducted accurately by attachment of the wearable biosensor on the human body directly. As sensing probes, GOx and lactate oxidase have also been introduced. The developed wearable biosensing patch system based on the MXene/Prussian blue composite could detect various target molecules in the sweat with high sensitivity in comparison with other reported carbon nanomaterial/Prussian blue-based biosensors. This biosensor exhibited the detection limit of 0.33 µM, detection sensitivity of 35.3 µA/mMcm^2^, and linear response from 10 µM to 1.5 mM for glucose detection. For lactate detection, it showed a detection limit of 0.67 µM, sensitivity of 11.4 µA/mMcm^2^, and linear response from 0 mM to 22 mM, a range that is included in the general human lactate concentration in the sweat. In addition, this biosensor retained its detection sensitivity over 15 days. This study shows that ultrasensitive enzymatic wearable electrochemical biosensors can be developed for practical application by using MXenes.

In addition to their use in enzymatic electrochemical biosensors, MXenes have been also used to develop electrochemical nucleic acid biosensors. In general, redox-generating molecules such as methylene blue and ferrocene are utilized to develop the electrochemical nucleic acid biosensors since, unlike metalloenzymes, nucleic acids do not possess redox characteristics. However, redox signals from widely used redox generating molecules are commonly low, making them difficult to be measured easily and sensitively. To overcome this limitation and to develop optimal electrochemical nucleic acid biosensors, researchers have extensively used MXenes to confer superior conductivity and as an effective matrix for nucleic acid immobilization.

For instance, the label-free electrochemical gliotoxin biosensor was developed by using nanocomplexes composed of the tetrahedral DNA nanostructure (TDN), MXene nanosheet, and horseradish peroxidase (HRP) [53]. To fabricate this biosensor, the MXene was synthesized by etching the Al layer of Ti_3_AlC_2_ using hydrogen fluoride (HF) solution, and the TDN was prepared by mixing equal quantities of the four different single-stranded DNA strands for the formation of the tetrahedral three-dimensional complex capturing probe molecule. Next, the prepared TDN was mixed with an aqueous solution of MXenes for production of the TDN/MXene complex, and the TDN/MXene complex was immobilized on the glassy carbon electrode (Figure 3c). The titanium element of the MXene nanosheet showed a strong chelation interaction with the phosphate groups of DNA. In addition, due to the tetrahedron structure of the prepared TDN, the TDN could be prevented from lying down for performing its role as the capturing probe efficiently. To investigate the gliotoxin detection performance of this biosensor, the researchers injected gliotoxin mixed with gliotoxin aptamer/signal probe into this biosensor. When gliotoxin was added to the gliotoxin aptamer/signal probe, the gliotoxin was strongly bound to the gliotoxin aptamer of the gliotoxin aptamer/signal probe. Then, the signal probe was detached from the gliotoxin aptamer and attached to the capturing probe of the TDN in the TDN/MXene complex. Finally, the gliotoxin detection performance of this biosensor could be evaluated by measurement of the signal from the signal probe bound to the TDN/MXene complex. Because of the high conductivity and flexibility of MXene nanosheets, this fabricated biosensor showed a highly sensitive limit of detection of 1.63 pg/mL and a detection range of 1.63 to 3260 pg/mL for gliotoxin by amperometric investigation, retaining its sensing property over 7 days. In addition to this study, an electrochemical multiple micro RNA (miRNA) biosensor was reported by using Au nanoparticles and MXene [62]. In this study, the MXene was used as the electrocatalyst for effective electrochemical signal enhancement, and Au nanoparticle was used to immobilize two different single-strand DNA (ssDNA) molecules modified with redox molecules (methylene blue and ferrocene), which were used as the sensing probes for multiple miRNA (miR-21 and miR-141) detection. This biosensor had a low assay time of 80 min and exhibited fourfold higher electrochemical signal than biosensors prepared without the MXene (detection sensitivity of 204 aM and 138 aM for miR-21 and miR-141 detection, respectively).

In addition to this example, a three-dimensional Au nanoparticle and MXene (Ti_3_C_2_) nanocomposite was developed for use as an electrochemical miRNA-155 biosensor [63]. The developed Au nanoparticle and MXene nanocomposite could facilitate redox signals from the methylene blue used as the redox probe in this research, thereby achieving high sensitivity (detection limit: 0.35 fM, linear response ranges from 1.0 fM to 10 nM). Moreover, MXenes have been recently combined with TMD nanomaterials, especially the MoS_2_, which is being studied and noted like MXenes. For example, the nanohybrid material composed of MoS_2_ and MXene (Ti_3_C_2_T_X_) was proposed to develop an electrochemical aptasensor for detection of thyroxine [64]. In this study, nanosheets of MoS_2_ and MXenes were hybridized on the screen-printed carbon electrode, and the three-dimensional Au nanostructures were electrochemically synthesized on the nanohybrid material layer to amplify the electrochemical signal derived from the detection of thyroxine. The thyroxine-specific RNA aptamer modified with ferrocene was employed on three-dimensional Au nanostructures synthesized on the nanohybrid material layer through Au–thiol binding. In the absence of thyroxine, the RNA aptamer existed as a folded structure, with the ferrocene located near the metallic surface. However, after addition of thyroxine, the structure of the RNA aptamer unfolded, which induced the faraway position of the ferrocene from the metallic surface and reduced the redox signal from the ferrocene. Using this sensing mechanism, a proposed biosensor based on the MoS_2_–MXene nanohybrid exhibited an ultralow limit of detection (0.39 pg/mL) and a wide linear response range (7.8 × 10^−1^ to 7.8 × 10^6^ pg/mL) because of the employment of nanohybrid materials and three-dimensional Au nanostructure simultaneously. In another study, the other type of nanohybrid material composed of the MoS_2_ and MXene (Ti_3_C_2_) was synthesized to develop the ultrasensitive miRNA-182 biosensor [65]. These studies suggest a novel direction combining MXenes with other novel nanomaterials to develop ultrasensitive electrochemical biosensors. In addition to studies in which biomolecules such as enzymes and nucleic acids were introduced as sensing probes, studies on the development of electrochemical biosensors without biomolecules are also in progress, including non-enzymatic electrochemical biosensors based on MXenes [66,67].

As discussed here, MXenes have huge potential for development of various types of electrochemical biosensors, on the basis of their exceptional properties suitable for electrochemical investigation. Much research is still underway to develop new types of MXenes, which show better performance with unique structures. On the basis of these studies, MXenes are expected to be used more actively for developing electrochemical biosensors with ultrasensitivity.

## 4. MXene-Based Fluorescent/Optical Biosensors

As seen in Section 2, MXenes possess not only electrical or electrochemical advantages such as high electrical conductivity, electrochemical activities, and large surface area, but also strong fluorescence efficiency [29,68]. In addition, MXenes show many useful characteristics suitable for fluorescent biosensors, including chemical stability, easy surface functionalization, fluorescent MQDs with high water solubility, dispersibility, and biocompatibility due to their high hydrophilicity; thus, development of fluorescent biosensors based on MXene nanosheets has gained momentum [41,69,70]. Novel synthesis techniques for easier and more efficient synthesis of MXene nanosheets and MQDs have been developed, and effective surface modification methods have been studied to functionalize the surface of MQDs to achieve high hydrophilicity and promote their fluorescent measurement efficiency under aqueous conditions [48,71].

**Figure 4:**
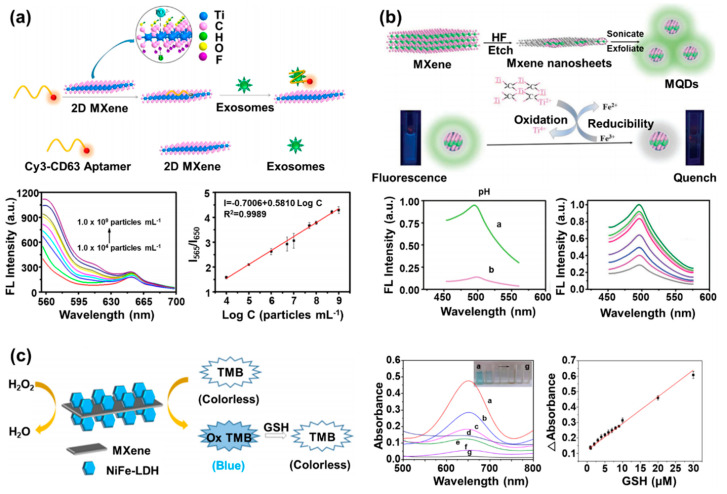
MXene-based fluorescent/optical biosensors. (**a**) Schematic image of the fluorescence resonance energy transfer (FRET)-based biosensing system based on the MXene nanosheets combined with Cy3-labeled CD63 aptamer and fluorescence signal of Cy3-labeled CD63 aptamer with different exosome concentrations and its linear calibration curve (reprinted with permission from [54]; copyright (2018) American Chemical Society). (**b**) Schematic images of the synthesis process of the MQDs and its Fe^3+^ detection mechanism, the fluorescence signal of the MQDs with or without 100 μM Fe^3+^, and the fluorescence signal of MQDs with different concentrations of Fe^3+^ (reprinted with permission from [55]; copyright (2020) The Royal Society of Chemistry). (**c**) Schematic image of the MXene nanosheet and the Ni, Fe layered double hydroxide (NiFe-LDH) (MXene/NiFe-LDH)-based colorimetric biosensor for glutathione (GSH) detection and absorbance spectrum of different configurations of MXene/NiFe-LDH in tetramethylbenzidine (TMB) and H_2_O_2_ with change of color and its linear calibration curve (reprinted with permission from [56]; copyright (2019) American Chemical Society).

On the basis of these efforts, optimal fluorescent biosensors based on the MXene nanocomposite have been developed, as summarized in Table 2. In most cases, the MXene nanosheets were used as quenching molecules that could block the emitted fluorescent signals from the fluorescent sensing probes before detection of the target molecules for operation of the fluorescent biosensors. Recently, fluorescence resonance energy transfer (FRET)-based biosensing systems have been developed to detect exosomes using Ti_3_C_2_ MXene nanosheets combined with the Cy3-labeled CD63 aptamer [54]. The CD63 is the exosome transmembrane protein. This exosome is one of the most attractive target molecules for containing important biomolecules such as the DNA and RNA secreted from the cell, allowing its use for non-destructive cellular monitoring [72]. To develop this biosensing system, researchers bound the Cy3 labeled-CD63 aptamer, which was employed as a sensing probe capable of binding with the exosome, to the synthesized MXene nanosheets via hydrogen bonds and metal-chelate interaction of the CD63 aptamer and MXene nanosheets (Figure 4a). In the absence of the exosome, the fluorescence signal of the Cy3 was quenched by the MXene nanosheets due to the proximity between the Cy3 and the MXene nanosheet. However, after addition of the exosome, the Cy3-labeled CD63 aptamer was detached from the MXene nanosheets via combination with the added exosome because the binding affinity of the Cy3-labeled CD63 aptamer and surface protein (CD63) of the exosome was stronger than that of the Cy3-labeled CD63 aptamer and MXene nanosheets. Therefore, the quenched signal of the Cy3 was restored, which allowed fluorescent detection of the exosome. By using this fluorescence-based sensing mechanism, this FRET-based biosensing system exhibited highly sensitive exosome sensing performance in a wide range from 10^4^ to 10^9^ exosomes/mL, with a low detection limit of 1.4 × 10^3^ exosomes/mL. This detection limit value was more than 1000 times lower than the value for conventional enzyme-linked immunosorbent assay (ELISA) methods. Furthermore, the fabricated FRET-based biosensing system maintained the exosome sensing function over 9 days, and even under a wide range of pH conditions (pH 6.4–8.4), it showed a stable fluorescence signal.

In another usage case, a fluorescent DNA biosensor was developed to diagnose the human papillomavirus (HPV) using fluorescein-labeled ssDNA (FAM-ssDNA) with Ti_3_C_2_ MXene nanosheets [73]. Like the previously discussed study, the fluorescence signal from the FAM was quenched when the FAM-ssDNA was immobilized on the MXene through the π–π interaction. However, when the target DNA, which was the partial sequence of the HPV gene, was added, the FAM-ssDNA formed double-stranded DNA (dsDNA) by hybridization with the target DNA and was released from the MXene. On releasing from the MXene, the fluorescence signal of the FAM was restored, and this fluorescent intensity was measured to analyze the concentration of the added target DNA. In addition, to enhance the detection sensitivity of the proposed biosensor, the researchers introduced exonuclease III (ExoIII), which cut the mononucleotides of dsDNA with 3′-hydroxyl terminus groups. In this cutting process, FAM was cleaved from dsDNA, and dsDNA was decomposed into ssDNA without FAM and target DNA. Then, the separated target DNA from the decomposed dsDNA could bind to the unreacted FAM-ssDNA still existing on the surface of MXene nanosheets again to induce substantial amplification of the fluorescent signal. Using this fluorescent DNA-sensing process, in the absence of ExoIII, the researchers measured the target DNA related to HPV, with a linear response range of 1.0 nM to 50 nM and a detection limit of 800 pM. However, when ExoIII was introduced, the detection sensitivity increased due to the fluorescent signal amplification phenomenon mentioned above. Thus, the detection ranges in which the target DNA was detected were widened (0.5 nM to 50 nM), and the detection limit was 100 pM. Similarly, the fluorescence-quenching property of MXene nanosheets has been utilized to demonstrate a fluorescent biosensing system for detection of various important biomolecules.

In addition to fluorescent biosensors developed using MXene nanosheets as a quenching material, MQDs have been developed and studied to develop these fluorescent biosensors by using the strong fluorescent emission property of the MQDs itself. For example, the fluorescent Ti_3_C_2_ MQD-based Fe^3+^ biosensor was developed in one study [55]. Fe^3+^ plays various roles in the physiological and pathological processes in the body, and when it is deficient or excessively accumulated in the body, it causes problems such as anemia, cancer, and dysfunction of organs [74]. Therefore, measurement of the exact concentration of Fe^3+^ in the body is essential. To develop a fluorescent biosensor for Fe^3+^ detection, the exfoliation of MXene nanosheets etched by hydrofluoric acid was performed by applying ultrasonic waves to synthesize MQDs with a size of 1.75 nm (Figure 4b). The synthesized MQDs were excited at 320 nm and generated the highest fluorescence emission signal at 410 nm, also showing stability within different solutions with a pH range of 6.4 to 8.4 and under high salt concentration (0.1 M of NaCl) conditions. Furthermore, the quantum yield of the synthesized MQDs was measured to be about 7.7%. The detection of Fe^3+^ was based on the following mechanisms. First, in the presence of the Fe^3+^, the internal filtering effect (IFE) occurred because the MQDs could not receive the excitation wavelength due to the interference of the Fe^3+^, which can absorb light in a range from 300 nm to 400 nm, which substantially overlaps with the excitation wavelength of the MQDs. Therefore, in the presence of both MQDs and Fe^3+^, some of the incoming excitation wavelength was absorbed by the Fe^3+^, not MQDs, reducing the fluorescence signal emitted from the MQDs. Second, by using the characteristics of the MQDs to reduce the Fe^3+^, the surface charge of the MQDs could be shifted to positive direction through the reduction of the Fe^3+^ on the surface of the MQDs. In this process, some of the Fe^2+^ were adsorbed on the MQD surface due to electrostatic interactions, which caused the fluorescence quenching of the MQDs. Because the MQDs were quenched due to the synergistic effect of the IFE of the Fe^3+^ and electrostatic interactions of the Fe^2+^, the researchers measured the concentration of Fe^3+^ in seawater and serum linearly in the range from 5 μM to 1000 μM through the analysis of the quenched fluorescence signal of the MQDs in proportional to concentration of added Fe^3+^. The detection limit of this biosensor was 310 nM, lower than the quality standard of drinking water (0.2 mg/L), which showed its possibility for practical application as the proposed fluorescent biosensor. Moreover, several samples containing Fe^3+^ were analyzed in triplicate, and the value of relative standard deviation (RSD) was calculated. The RSD values of 1.1% and 1.2% were obtained at 10 μM and 250 μM of Fe^3+^, respectively. This result indicates that the Fe^3+^ detection method using the fabricated Ti_3_C_2_ MQD-based Fe^3+^ biosensor had excellent reproducibility. Another similar example is the development of the fluorescent polylysine-coated Ti_3_C_2_ MQD (PL-MQD)-based biosensor for detection of cytochrome c (Cyt c) and trypsin [75]. Cyt c and trypsin are the important proteins in the cell death process and involve diseases such as pancreatitis, intestinal obstruction, and cystic fibrosis [76,77]. After the ultrasonic waves were applied to the MXene nanosheets for formation of the MXene nanoparticles, the PL was added and reacted with prepared MXene nanoparticles by the hydrothermal method for synthesis of the PL-MQDs. With the introduction of the PL onto the MQD surface, synthesized PL-MQDs showed a high quantum yield of about 22%. In addition, when excited by a wavelength of 330 nm, the PL-MQDs exhibited the strong fluorescent emission at 415 nm. Furthermore, synthesized PL-MQDs showed the stable fluorescence signal in a wide range from pH 1 to pH 13, and the decrease of fluorescence signal was hardly observed even over 1 month. When the Cyt c and PL-MQD existed together, the IFE effect was noted due to the broad absorbance spectrum (from 400 nm to 600 nm) of Cyt c. As a result, the fluorescence signal of the PL-MQDs decreased in proportion to the amount of the added Cyt c. The concentration of Cyt c was measured linearly from 0.2 μM to 40 μM, and the detection limit was 20.5 nM. Next, trypsin was added to a solution in which the PL-MQDs and 40 μM of Cyt c was mixed. The trypsin hydrolyzed a Cyt c into small pieces of peptide fragments by a catalytic reaction, and the fluorescence signal of the PL-MQDs gradually recovered because of the disappearance of the Cyt c. Accordingly, the fluorescence signal intensity of the PL-MQDs increased in proportion to the amount of the added trypsin. The concentration of trypsin was measured linearly in the range of 0.5 μg/mL to 80 μg/mL, and the detection limit was 0.1 μg/mL.

In addition to MXene-based fluorescent biosensors, colorimetric optical biosensors based on MXene nanocomposites have been studied extensively due to their simplicity, rapid usage, low cost, and sensitive detection by naked eyes, which make them suitable for point-of-care testing [78,79,80,81]. For development of colorimetric optical biosensors, the peroxidase-like properties of MXenes are utilized. For example, the heterostructure composed of the Ti_3_C_2_T_x_ MXene nanosheet and the Ni, Fe layered double hydroxide (NiFe-LDH) (MXene/NiFe-LDH) was developed for colorimetric detection of glutathione (GSH) (Figure 4c) [56]. Both the MXene nanosheets and NiFe-LDH showed peroxidase-like activity. However, when the MXene nanosheets and NiFe-LDH were combined and formed the MXene/NiFe-LDH, the combination exhibited a higher catalytic property than the catalytic properties of the MXene nanosheets or NiFe-LDH alone. This result was achieved because of the increase in the surface area accessible to ions and the acceleration of the electron transfer rate by surface conjugation of the MXene nanosheets and NiFe-LDH. The concentration of GSH was measured by adding GSH to an aqueous solution containing the MXene/NiFe-LDH, 3,3′,5,5′-tetramethylbenzidine (TMB), and H_2_O_2_. The MXene/NiFe-LDH oxidized the TMB in the presence of H_2_O_2_, and the blue color of TMB was changed to the transparent color of the oxidized TMB. However, after addition of GSH, the oxidized TMB oxidized the GSH, and the TMB itself was reduced to display the blue color again. On the basis of this colorimetric change, the detection of GSH was performed through the assessment of the restored blue color intensity of the TMB (the linear response from 0.9 μM to 30 μM concentration and detection limit of 84 nM). In addition, high reliability of the MXene/NiFe-LDH-based detection method was confirmed by showing a recovery rate in the range of 92% to 107% and an RSD value of 0.93% to 2.36% with the GSH concentration of 1 μM, 10 μM, and 20 μM in human serum samples.

As discussed in this section, MXenes are widely applicable to the development of fluorescent/optical biosensors with their unique surface chemical characteristics. Although there are some limitations to be solved, such as complexity of the synthesis process and the low oxidative stability. However, since many studies are underway to control the properties of MXene accurately, it is expected that there will be more advances in development of the Mexen-based fluorescent/optical biosensors beyond these limitations in the near future.

## 5. Conclusions and Future Perspectives

Numerous nanomaterials have been developed, synthesized, and utilized in the field of biosensors to harness their exceptional inherent advantages for achievement of high sensitivity and selectivity. The intrinsic characteristics of nanomaterials, such as their high conductivity and plasmonic and fluorescent properties, make them suitable for the development of advanced biosensors. Among various recently reported nanomaterials, MXenes have attracted much attention because their exceptional properties make them suitable for the development of advanced biosensors. Accordingly, since they were first reported, MXenes have been extensively studied for their potential for biosensor development. Moreover, recent studies on the development of biosensors based on the MXene nanocomposite have verified that MXenes are the best candidate among various nanomaterials for the development of various types of biosensors, including electrochemical, fluorescent, and optical biosensors, and still have huge potential for the development of next-generation biosensors such as wearable biosensing systems.

Considering these points of view, we have described the exceptional properties of MXene and recently developed biosensors on the basis of the MXene nanocomposite. For this, we provided the synthesis methods and properties of MXene and its application for developing the electrochemical and fluorescent/optical biosensors. As discussed here, MXene improves the performance of biosensors and helps in the development of advanced biosensors. Nevertheless, numerous obstacles remain for commercial utilization of these MXene-based biosensors, such as the reproducibility of these biosensors and the scope for their massive production. However, continuing research for the development of new synthesis methods or the novel structures of MXenes will form the basis for commercialization of various MXene nanocomposites and development of commercially available MXene-based biosensors. Moreover, these follow-up studies are expected to suggest a more effective conjugation way between MXene and other nanomaterials, contributing to maximizing the inherent properties of novel MXene nanocomposites to be developed in the near future. In conclusion, we strongly believe that this review can provide the creative direction for efficient utilization of MXene nanocomposites to develop advanced biosensors with high sensitivity and selectivity.

## Figures and Tables

**Table 1:** Table of the studies for synthesis of MXenes using different etching solutions.

MAX Phases	Etching Solution and Condition	MXenes	Characteristics	Reference
Ti_3_AlC_2_ powder	50% HF, room temperature, 2 h	Ti_3_C_2_ (nanosheet)	Excellent electrical conductivity and hydrophilic	[37]
Nb_2_AlC powder	40% HF, 60 °C, 90 h	Nb_2_CT_x_ (nanosheet)	Excellent electrical conductivity	[38]
Ti_3_AlC_2_ powder	LiF in 6 M HCl, 35 °C, 24 h	Ti_3_C_2_Tx (nanosheet)	Defect structure and excellent electrical conductivity	[39]
Ti_3_AlC_2_ powder	1 M bifluoride (NaHF_2_, KHF_2_, NH_4_HF_2_), 60 °C, 8 h	Ti_3_C_2_ (nanosheet)	Large interplanar spacing	[40]
Ti_3_AlC_2_ powder	49% HF, toom temperature, 24 h and hydrothermal reaction with DMF, ethanol, DMSO	Ti_3_C_2_T_x_ (quantum dot)	Excellent optical properties and biocompatibility	[41]

**Table 2:** Summary of the studies in which efficient biosensors were prepared using the MXene nanocomposite.

Biosensors Based on the MXene Nanocomposite							
Type	Composition	Sensing Probe	Target	Utilized Technique	Sensitivity	Stability	Reference
MXene-based electrochemical biosensors	Ti_3_C_2_T_x_ MXene/graphene/GOx/glassy carbon	GOx	Glucose	Cyclic voltammetry	Detection sensitivity: 12.10 μA/mM	Negligible current decrease over 300 scanning cycles	[51]
	MXene/CNT/Prussian blue/enzymes/carbon fiber	GOx and lactate oxidase	Glucose and lactate in the sweat	Chrono amperometry	Detection limit: 0.33 µM, detection sensitivity: 35.3 µA/mMcm^2^ for glucose, detection limit: 0.67 µM, detection sensitivity: 11.4 µA/mMcm^2^ for lactate	Retention of its detection sensitivity over 15 days	[52]
	Ti_3_C_2_ MXene/TDN/HRP/gliotoxin aptamer/signal probe/glassy carbon	Gliotoxin aptamer/signal probe	Glio toxin	Amperometry	Limit of detection: 1.63 pg/mL, 5 Pm	Retention of its current response over 7 days	[53]
MXene-based fluorescent/optical biosensors	MXene nanosheets/Cy3-labeled CD63 aptamer	Cy3-labeled CD63 aptamer	Exo some	Fluorescence	Detection limit: 1.4 × 10^3^ exosomes/mL	Stable signal in pH6.4–8.4 range and retention of signal over 10 days	[54]
	MQDs/Fe^3+^	MQDs	Fe^3+^	Fluorescence	Limit of detection: 310 nM	Stable signal in pH 6.4–8.4 range and RSD for 10 and 250 μM of Fe^3+^ was 1.1% and 1.2%	[55]
	MXene/NiFe-LDH/ TMB/H_2_O_2_	TMB	GSH	Colorimetry	Detection limit: 84 nM	-	[56]
